# Faecal microbiota of domestic cats fed raw whole chicks *v.* an extruded chicken-based diet*

**DOI:** 10.1017/jns.2014.21

**Published:** 2014-09-25

**Authors:** K. R. Kerr, S. E. Dowd, K. S. Swanson

**Affiliations:** 1 Department of Animal Sciences and Division of Nutritional Sciences, University of Illinois at Urbana-Champaign, IL, USA; 2 MR DNA (Molecular Research LP), Shallowater, TX, USA

**Keywords:** Feline nutrition, Gut microbiota, Raw diet, CHI, 1–3-d-old chicks, CP, crude protein, EXT, chicken-based extruded diet

## Abstract

Extruded cat foods differ greatly in macronutrient distribution compared with wild-type diets (i.e. small mammals, reptiles, birds and insects). Based on the literature, this variability likely impacts faecal microbial populations. A completely randomised design was utilised to test the impacts of two dietary treatments on faecal microbial populations: (1) chicken-based extruded diet (EXT; *n* 3 cats) and (2) raw 1–3-d-old chicks (CHI; *n* 5 cats). Cats were adapted to diets for 10 d. Bacterial DNA was isolated from faecal samples and amplicons of the 16S rRNA V4–V6 region were generated and analysed by 454 pyrosequencing. Faeces of cats fed CHI had greater (*P* < 0·05) proportions of the following bacterial genera: unidentified Lachnospiraceae (15 *v.* 5 %), *Peptococcus* (9 *v.* 3 %) and *Pseudobutyrivibrio* (4 *v.* 1 %). Faeces of cats fed EXT had greater (*P* < 0·05) proportions of *Faecalibacterium* (1·0 *v.* 0·2 %) and *Succinivibrio* (1·2 *v.* < 0·1 %). Five genera, including *Lactobacillus* and *Bifidobacterium*, were present in a majority of samples (two to three out of three) from cats fed EXT, but were not detected in the samples (zero of five) for cats fed CHI. These shifts in faecal bacterial populations compared with feeding a whole-prey diet may impact the functional capacities of the microbiota and its interaction with the host. Further research is warranted to determine the impacts of these shifts on long-term health of domestic cats.

In the wild, feral cats typically eat small mammals, reptiles, birds and insects. It is often not possible to mimic natural feeding behaviours of feral cats. Extruded diets have been the traditional alternative fed to domestic cats. Commercial chicken-based extruded diets (EXT) have complex diet formulations, including protein, fat, carbohydrate, fibre, vitamin and mineral ingredients. Association of American Feed Control Officials (AAFCO)^(^^1^^)^ recommend minimum concentrations of 26 % crude protein (DM basis) and 9 % fat DM basis. If these minimum concentrations are targeted by formulators, commercial feline EXT may contain up to 50–55 % carbohydrates. Plantinga *et al.*^(^^2^^)^ estimated the diet of feral cats expressed on a DM basis would contain 63 % crude protein (CP), 23 % fat and 2·8 % nitrogen-free extract (i.e. digestible carbohydrate).

Given their carnivorous nature, it has been hypothesised that the protein:carbohydrate ratio of feline diets is important for feline health (i.e. obesity, feline diabetes and gut microbiota)^(^^3^^–^^6^^)^. As lower bacterial diversity and great shifts in commensal bacteria are often present in inflammatory bowel diseases, it suggests that a balanced gut microbiota is important for maintaining host health^(^^7^^–^^9^^)^. Several studies have examined the impact of dietary alterations on faecal microbial populations in cats^(^^3^^–^^5^^,^^10^^–^^13^^)^; however, very few have examined the microbial population of cats fed a ‘wild-type’ diet^(^^12^^,^^13^^)^. Commercially available whole prey may be more similar to the feral cat diet. For example, commercially available 1–3-d-old chicks (CHI) are approximately 72–76 % CP, 16–20 % fat and <5 % nitrogen-free extract^(^^14^^,^^15^^)^. Previous studies have shown that extruded and whole-prey diets differ in digestibility as well as macronutrient composition^(^^14^^–^^17^^)^, and this may alter the fermentable substrates that are available to the gastrointestinal microbiota for fermentation^(^^18^^,^^19^^)^. The objective of the present study was to compare the faecal microbiota of cats fed an EXT chicken-based diet to those fed commercially available whole CHI.

## Experimental methods

### Study design

The animal protocol was approved by the University of Illinois Animal Care and Use Committee. Faecal samples were collected from neutered male domestic cats (mean age = 5·7 years; body condition score 4·5–5·5 of 9). A completely randomised design was utilised to test the impacts of two dietary treatments (Table 1): (1) EXT (*n* 3 cats; P & G Petcare); and (2) raw CHI (*n* 5 cats; Rodent Pro). The raw chicks were frozen (−20°C) upon arrival, and thawed in the refrigerator for 24 h prior to feeding. Fresh water was available *ad libitum*. A computer was used to randomly allot cats to treatment. Cats were adapted to diets for 10 d, prior to fresh faecal collection (<15 min from defection). Faecal samples were stored at −80°C until DNA extraction.

**Table 1.** Chemical composition of CHI and an EXT fed to domestic cats*

Item	CHI	EXT†
DM (%)	24·2	95·4
Organic matter (% DM)	91·1	92·8
Crude protein (% DM)	71·4	38·9
Acid-hydrolysed fat (% DM)	20·0	14·4
Gross energy (kcal/g DM)	5·9	5·2

* CHI, 1–3-d-old chicks (Rodent Pro); EXT, extruded chicken-based diet (P & G Petcare).

†Ingredient composition of EXT as reported by manufacturer: chicken, chicken by-product meal, maize meal, maize grits, dried beet pulp, poultry by-product meal, natural flavour, dried egg product, brewers dried yeast, sodium bisulphate, potassium chloride, fructooligosaccharides, animal fat (preserved with mixed tocopherols, a source of vitamin E), fish oil (preserved with mixed tocopherols, a source of vitamin E), DL-methionine, choline chloride, calcium carbonate, vitamins (vitamin E supplement, niacin, ascorbic acid, vitamin A acetate, calcium pantothenate, biotin, thiamine mononitrate (source of vitamin B_1_), pyridoxine hydrochloride (source of vitamin B_6_), vitamin B_12_ supplement, riboflavin supplement (source of vitamin B_2_), inositol, vitamin D_3_ supplement and folic acid), taurine, minerals (zinc oxide, manganese sulphate, copper sulphate, potassium iodide and cobalt carbonate) and rosemary extract.

### Sample analysis

Faecal bacterial DNA was isolated according to procedures described previously^(^^20^^)^ using the MO BIO PowerSoil™ Kit (MO BIO Laboratories). Amplification of a 600 bp sequence of the V4–V6 variable regions of the 16S rRNA gene was done using barcoded primers as previously described^(^^21^^)^. PCR amplicons were further purified utilising AMPure XP beads (Beckman-Coulter Inc.). Amplicons were combined in equimolar ratios to create a DNA pool that was used for pyrosequencing. DNA quality of amplicon pools was assessed before pyrosequencing using a 2100 Bioanalyzer (Agilent Technologies). Pyrosequencing was performed at the W. M. Keck Center for Biotechnology at the University of Illinois utilising a 454 Genome Sequencer and FLX titanium reagents (Roche Applied Science).

### Data analysis

High-quality (quality value >25) sequence data derived from the sequencing process was processed using a proprietary analysis pipeline (www.mrdnalab.com) and as described previously^(^^22^^)^.

### Statistical analysis

Sequence percentages at each taxonomic level were analysed using the Mixed models procedure of SAS (version 9.3; SAS Institute). The fixed effect of diet was tested. Means were separated for treatments using a Fisher-protected least significant difference with Tukey's adjustment. Results are reported as least-squares means with *P* ≤ 0·05 defined as significant and *P* ≤ 0·10 as trends for treatment effects.

## Results

Regardless of dietary treatment, Firmicutes (62–88 % of all sequences) was the predominant bacterial phylum in cat faeces (data not shown). Fusobacteria (0·2–17 % of all sequences), Proteobacteria (2–16 % of all sequences), Actinobaceria (1·4–18 % of all sequences), Tenericutes (1·4–9 % of all sequences) and Bacteroidetes (0–3 % of all sequences) also were predominant phyla present (data not shown). The proportion of Bacteroidetes was greater (*P* = 0·03) in faeces of cats fed EXT (1·6 % of all sequences) than those fed CHI (0·2 % of all sequences; data not shown).

Proportions of genera, however, depended on dietary treatment (Table 2). The predominant genera in faeces of cats fed CHI were *Clostridium* (11–25 % of sequences), *Blautia* (4–19 % of sequences), unidentified Lachnospiraceae (14–16 % of sequences), *Peptococcus* (7–13 % of sequences), *Fusobacterium* (4–13 % of sequences), *Ruminococcus* (2–9 % of sequences) and *Collinsella* (2–8 % of sequences). The predominant genera in faeces of cats fed EXT were *Megamonas* (2–28 % of sequences), *Megasphaera* (0·01–26 % of sequences), *Blautia* (10–16 % of sequences), *Collinsella* (1–16 % of sequences), *Lactobacillus* (0·2–14 % of sequences), *Clostridium* (8–12 % of sequences) and unidentified Lachnospiraceae (4–7 % of sequences).

**Table 2.** Predominant bacterial genera (expressed as percentage of sequences) in faeces of domestic cats fed CHI (*n* 5) or EXT (*n* 3)*

Phylum	Family	Genus	CHI	EXT	sem	*P* value
Actinobacteria	Bifidobacteriaceae	*Bifidobacterium*	ND†	0·3	–	–
	Bifidobacteriaceae	*Collinsella*	4·9	6·9	17·0	0·94
	Coriobacteriaceae	*Slackia*	0·1	<0·1	<0·1	0·06
		Unidentified genera	0·1	0·5	0·1	0·08
Firmicutes	Acidaminococcaeae	*Phascolarctobacterium*	0·6	1·3	0·2	0·09
	Clostridiaceae	*Clostridium*	16·3	10·5	2·3	0·12
	Enterococcaeae	*Enterococcus*	1·0	0·3	0·3	0·13
	Erysipelotrichaceae	*Allobaculum*	0·8	0·1	0·2	0·06
		Unidentified genera	0·7	<0·1	0·3	0·22
	Eubacteriaceae	*Eubacterium*	5·8	2·5	1·1	0·09
	Lachnospiraceae	*Blautia*	9·7	12·3	2·7	0·51
		*Coprococcus*	2·3	0·6	1·0	0·30
		*Psuedobutyrivibrio*	4·0	1·0	0·8	0·04
		*Roseburia*	1·2	0·3	0·5	0·25
		Unidentified genera	15·2	5·3	0·5	<0·01
	Lactobacilliaceae	*Lactobacillus*	ND	7·6	–	–
	Oscillospiraceae	*Oscillibacter*	0·3	<0·1	0·1	0·09
	Peptococcaeae	*Peptococcus*	9·2	3·2	1·0	<0·01
	Peptostreptococcaeae	*Peptostreptococcus*	0·7	<0·1	0·5	0·43
	Ruminococcaceae	*Anaerotruncus*	2·3	1·3	0·4	0·13
		*Faecalibacterium*	0·2	1·0	0·2	0·02
		*Ruminococcus*	4·3	2·2	1·2	0·28
		Unidentified genera	2·4	1·1	0·4	0·06
	Veillonellaceae	*Megamonas*	<0·1	11·9	4·1	0·09
Fusobacteria	Fusobacteriaceae	*Fusobacterium*	8·7	5·9	15·1	0·83
Proteobacteria	Campylobacteraceae	*Campylobacter*	2·4	0·6	2·2	0·59
	Enterobacteriaceae	*Shigella*	2·7	0·7	1·2	0·29
	Succinivibrionaceae	*Anaerobiospirillum*	0·8	1·2	0·6	0·64
		*Succinivibrio*	<0·1	1·2	0·2	<0·01

* CHI, 1–3-d-old chicks (My Pet Carnivore); EXT, extruded chicken-based diet (P&G Petcare).

†ND, not detected.

Four genera were present in a majority of samples (*n* 5) for cats fed CHI (*Holdemania* (three of five; 0–0·5 % of sequences), *Escherichia* (four of five; 0–0·2 % of sequences), *Marvinbyantia* (five of five; 0·01–0·1 % of sequences) and *Acetanaerobacterium* (three of five; 0–0·02 % of sequences)), but were not detected in the samples for cats fed EXT (data not shown). Five genera were present in a majority of samples (*n* 3) for cats fed EXT (*Megasphaera* (three of three; 0·01–26 % of sequences), *Lactobacillus* (three of three; 0·2–14 % of sequences), *Prevotella* (three of three; 0·1–2·6 % of sequences), *Subdoligranulum* (two of three; 0–1·4 % of sequences) and *Bifidobacterium* (two of three; 0–0·8 % of sequences), but were not detected in the samples for cats fed CHI (data not shown). Cats fed CHI had greater (*P* < 0·05) *Psuedobutyrivibrio*, unidentified Lachnospiraceae and *Peptococcus* populations and tended to have greater (*P* < 0·10) *Slackia*, *Allobaculum*, *Eubacterium*, *Oscillibacter* and unidentified Ruminococcaceae populations. In contrast, cats fed CHI had lower (*P* < 0·05) *Faecalibacterium* and *Succinivibrio* populations and tended to have lower (*P* < 0·10) *Phascolarctobacterium*, *Megamonas* and unidentified Coriobacteriaceae populations.

## Discussion

We identified a significant shift in the faecal bacteria of cats fed CHI *v.* EXT. As these diets contained ingredient and nutrient differences, differences in proportions of bacterial populations can only be attributed to the treatments as a whole. To our knowledge, most of the studies investigating the effects of diet on bacterial composition utilising next-generation sequencing in cats have examined the effects of commercial dry^(^^5^^,^^10^^,^^11^^)^ and canned diets^(^^3^^,^^4^^)^. Only preliminary data for the differences in bacterial composition between a raw meat and kibbled diets fed to dogs have been reported^(^^23^^,^^24^^)^. No data have been reported for a whole-prey diet type in either cats or dogs; however, in a companion paper, we also present the microbial populations of cats fed whole and ground chicks and the effects of clinically confirmed symptomatic salmonellosis^(^^13^^)^.

Bermingham *et al.*^(^^4^^)^ reported increased faecal proportions of *Lactobacillus* (32 *v.* 0·1 % of sequences) and *Megasphaera* (23 *v.* <0·1 % of sequences) in the faecal microbiota of cats fed a commercial dry diet (i.e. lower protein, higher nitrogen-free extract; CP = 33 %, DM basis; fat = 11 %, DM basis) compared with those maintained on a commercial wet diet (i.e. higher protein, lower nitrogen-free extract; CP = 42 %, DM basis; fat = 42 %, DM basis). Hooda *et al.*^(^^5^^)^ reported increased faecal proportions of *Megasphaera* (18–33 *v.* <0·1–0·1 % of sequences), *Subdoligranulum* (2–6 *v.* 0·1–0·3 % of sequences) and *Bifidobacterium* (12–21 *v.* < 0·1–0·1 % of sequences) in kittens fed a moderate protein-moderate carbohydrate diet (CP = 34 %, DM basis; fat = 19 %, DM basis) compared with those fed a high-protein, low-carbohydrate diet (CP = 53 %, DM basis; fat = 24 %, DM basis). Beloshapka *et al.*^(^^23^^)^ reported increased faecal proportions of *Faecalibacterium* (10 *v.* 0·3 % of sequences), *Lactobacillus* (9 *v.* < 0·1 % of sequences) and *Prevotella* (9 *v.* 0·2 % sequences) for dogs fed an EXT compared with raw-meat-based diets. Although these studies also reported other differences not observed herein, those listed here are similar to our data, and indicated that the protein:carbohydrate ratio may impact these genera. However, both Bermingham *et al.*^(^^4^^)^ and Hooda *et al.*^(^^5^^)^ reported decreased faecal proportions of *Faecalibacterium* (<0·1 *v.* 0·5 % of sequence, and 0·1–2 *v.* 5–7 % of sequences, respectively) for the lower-protein *v.* higher-protein diet, which are contrary to our results and those reported by Beloshapka *et al.*^(^^23^^)^.

Another aspect of diet that can impact microbial populations is dietary fibre. Although it has been recognised that animal tissues provide substrate for fermentation (i.e. animal fibre^(^^18^^,^^19^^)^), their role in gut health has not been fully elucidated, and little is known about their impacts on microbial populations. The EXT diet tested herein included multiple ingredients that would contribute to the dietary fibre fraction, including beet pulp and fructooligosaccharides, which likely contributed to the differences in microbial populations. *Lactobacillus*, *Bifidobacterium* and *Faecalibacterium* species are generally considered beneficial bacteria and are often targeted with dietary fibre and prebiotic inclusions. Middelbos *et al.*^(^^25^^)^ reported increased faecal proportions of *Faecalibacterium* (30 *v.* 9 % of sequences) in dogs fed a diet containing 7·5 % beet pulp fibre (total dietary fibre = 4·5 %, DM basis) compared with 0 % supplemental fibre (total dietary fibre = 1·4 %, DM basis). Fructooligosaccharides are rapidly fermented and serve as a source of soluble, prebiotic fibre^(^^26^^,^^27^^)^. Several studies in cats and dogs have reported that fructooligosaccharides exert a prebiotic effect in the colon, stimulating the growth of *Bifidobacterium* spp., *Lactobacillus* spp. or both^(^^10^^,^^28^^)^. These studies are consistent with the results reported herein.

The present study had limitations, including our sampling protocol, number of animals and the nature of the diets tested. First, baseline samples were not collected before dietary treatments were administered. Thus, while differences due to diet were identified, we were unable to identify microbiome shifts from baseline. Second, given the low number of animals studied, our statistical power was low and our ability to translate the data to larger cat populations was limited. Finally, because the diets were greatly different in terms of nutrient composition and physical form, microbiome differences could not be attributed to any single factor or nutrient, but only the entire diet as a whole.

To conclude, there is growing evidence that the proportions of gastrointestinal microbes are altered in some disease states, including cancer, gastrointestinal diseases and metabolic diseases^(^^29^^–^^31^^)^. Given the potential of diet to modulate microbial populations, diet therapies may play a role in their treatment. However, it is unclear if this dysbiosis is causative or symptomatic of these disease states. Additionally, there is little data available regarding microbial populations, dysbiosis and disease states for cats^(^^8^^,^^13^^)^. The present study has highlighted some interesting differences in gastrointestinal microbes of cats eating extruded *v.* raw diets. More research is needed, however, to determine the long-term impacts of the alterations in the proportions of faecal microbial populations and the health of domestic cats.
